# Evaluation of Hypoglycemic Activity and Sub-Acute Toxicity of the Novel Biochanin A–Chromium(III) Complex

**DOI:** 10.3390/molecules27185786

**Published:** 2022-09-07

**Authors:** Pengshou Li, Mengdan Li, Xinhua Lou, Baosheng Zhao, Qixiang Ma, Yumiao Bian, Xiliang Mi

**Affiliations:** 1 School of Food and Drug, Luoyang Normal University, Luoyang 471934, China; 2 Beijing Academy of Traditional Chinese Medicine, Beijing University of Chinese Medicine, Beijing 100029, China; 3 Cancer Institute, Fudan University Cancer Hospital and Cancer Metabolism Laboratory, Institutes of Biomedical Sciences, Fudan University, Shanghai 200032, China

**Keywords:** biochanin A, chromium complex, hypoglycemic activity, sub-acute toxicity

## Abstract

The novel biochanin A–chromium(III) complex was synthesized by chelating chromium with biochanin A (BCA). The structure of the complex was determined and the complex ([CrBCA_3_]) was composed of chromium(III) and three ligands, and the chromium content was 55 μg/mg. The hypoglycemic activity of the complex was studied in db/db mice and C57 mice. The sub-acute toxicity test of the complex was carried out by the maximum limit method in KM mice. The hypoglycemic activity showed that the complex could reduce the weight of db/db mice and lower the fasting blood glucose and random blood glucose levels. The complex also improved the organ index, oral glucose tolerance test (OGTT) and insulin tolerance test (ITT) results of db/db mice, and some of the indicators were similar to those of the positive control group after treatment with the complex. The histopathology study showed significant improvements in the liver, kidney, pancreas and skeletal muscle compared with the diabetes model group. The complex also showed a significant improvement in serum biochemical indices and antioxidant enzyme activities, as well as glycogen levels. The sub-acute toxicity study showed that the complex did not cause death or any dangerous symptoms during the study. In addition, the sub-acute toxicity study showed that the complex had no significant effect on the serum biochemical indices, antioxidant capacity and organs of normal mice. This study showed that [CrBCA_3_] had good hypoglycemic activity in vivo and had no sub-acute toxicity. This work provides an important reference for the development of functional hypoglycemic foods or drugs.

## 1. Introduction

Diabetes is one of the most important public health problems in the world [1]. It is characterized by hyperglycemia, hyperlipidemia and insulin resistance [1]. As a result of the continuous deterioration of pancreatic cell function, long-term blood glucose control is difficult to achieve with current drug therapy [2]. Treatment of diabetes is one component, but prevention is the key.

Therefore, dietary supplements, which can improve the carbohydrate balance, are very popular. Chromium(III) is a beneficial trace element for the human body. Chromium compounds exhibited hypoglycemic activity and could improve insulin sensitivity, lipid metabolism and insulin resistance [3,4,5,6]. Organic chromium compounds such as chromium picolinate, chromium nicotinate and chromium yeast have been widely used in functional foods and nutritional supplements. Chromium picolinate is the most widely used. However, chromium picolinate has been found to cause DNA toxicity, cytotoxicity and reproductive toxicity [7,8], and its toxicity was mainly due to its ligand [9,10,11]. Therefore, the development of some new organic chromium(III) complexes or functional chromium(III) food supplements in type II diabetes is a good solution, and the ligand plays an important role in improving the bioactivity and safety of chromium(III) complexes. Many types of chromium complexes have been obtained by changing the ligand. Such complexes include organic acid chromium complexes [12,13], organic amine chromium complexes [14,15], amino acid chromium complexes [16], polysaccharide chromium complexes [17,18], oligosaccharide chromium complexes [19], monosaccharide chromium complexes [20], flavonoid chromium complexes [21], peptide chromium complexes [22] and polyphenol chromium complexes [23], etc. Some of the chromium complexes, such as chromium malate, have been studied extensively and have good application value due to their hypoglycemic activity [24].

Biochanin A (BCA) is the main active ingredient in *Cicer arietinum* L. (Fabaceae). BCA is an isoflavonoid, with many pharmacological effects [25]. BCA has anti-tumor effects and can inhibit breast cancer [26,27] and prostate cancer [28,29]. BCA also has anti-osteoporosis [30], antioxidation [31], anti-inflammation [32] and anti-bacterial effects [33], and it influences the nervous system [34]. BCA may induce hypoglycemic effects by stimulating islet B cells to secrete insulin, enhancing glycolytic enzymes and reducing the activity of gluconeogenesis enzymes [35]. Other studies showed that BCA, as an effective peroxisome proliferator-activated receptor α (PPARα) agonist, can improve the arteriosclerosis of diabetic complications by activating PPARα, which is effective in the treatment of diabetes [36]. The present research indicated that BCA also displayed good effects on type 2 diabetes, which may be linked to SIRT1 expression [37].

At present, animal models of type 2 diabetes include spontaneous type 2 diabetes animal models, induced type 2 diabetes animal models and genetic engineering type 2 diabetes animal models [38]. In this study, we used a spontaneous type 2 diabetes animal model—db/db mice. db/db mice have a defect in the leptin receptor gene that causes spontaneous type 2 diabetes, and db/db mice are characterized by obesity, hyperglycemia, dyslipidemia and hyperinsulinemia [39]. Their physiological and behavioral characteristics are highly similar to those of human type 2 diabetes, so it is an ideal animal model of human type 2 diabetes [40,41].

In this study, we selected BCA as the ligand, and formed a complex with chromium(III). To study its hypoglycemic activity, toxicity and side effects, we expected to obtain organic chromium complexes with good hypoglycemic activity and low toxicity and side effects. This study expands the scope of chromium complexes and provides a scientific basis for the research and development of chromium complexes of BCA as a hypoglycemic functional food and nutrient supplement.

## 2. Results

### 2.1. Synthesis and Structural Characterization of [CrBCA_3_]

#### 2.1.1. Physicochemical Properties, Elemental Analysis and Mass Spectrometry Analysis

**Figure 1:**
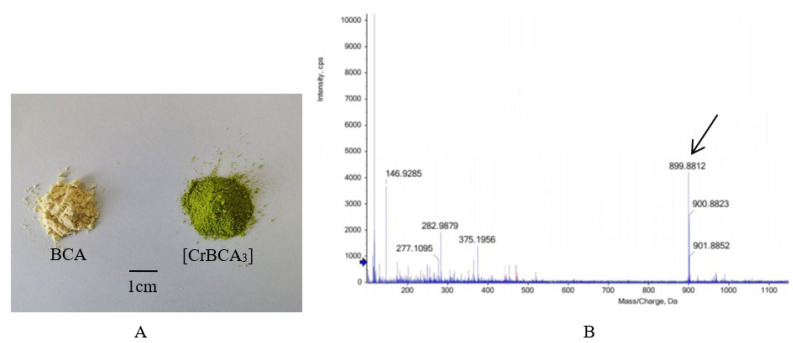
Sample condition and HRMS. (**A**) BCA and [CrBCA_3_]. (**B**) Mass spectrum of [CrBCA_3_].

The chromium complex [CrBCA_3_] was in the form of a grass-green powder that could be dissolved in dimethyl sulfoxide, dimethyl formamide, ethanol, methanol and ethyl acetate (Figure 1A). The complex was insoluble in water and CCl_4_. The results of elemental analysis and MS data are shown in Table 1 and Figure 1B. From the results, it could be seen that the actual measured results were consistent with the final calculated results.

#### 2.1.2. Thermogravimetry (TG) and Differential Thermal Analysis (DTA)

Under the condition of 10 °C/min heating rate in flowing air, the weight loss of the complex in the first stage occurred at approximately 85 °C. The weight loss rate of the complex was 3.86%, which was equivalent to the weight loss of two water molecules (the theoretical weight loss rate was 3.84%). There was a small endothermic peak (83 °C) on the DTA curve, indicating that the complex had two molecules of crystal water. The second stage was the oxidation and decomposition of the complex. At 325–425 °C, the weight loss rate was 90.59%, which was equivalent to the weight loss of three ligand molecules (the theoretical weight loss rate was 90.61%). The corresponding DTA curve showed a large exothermic peak, which was the oxidation peak of the complex (383 °C), indicating that the three ligand molecules decomposed rapidly and chromium was oxidized in air. The residue obtained after the decomposition of the complex was an oxide of chromium (Cr_2_O_3_).

#### 2.1.3. Infrared Spectrum Analysis

**Figure 2:**
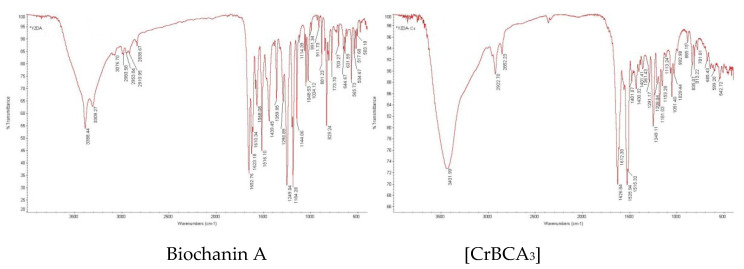
IR spectra of BCA and [CrBCA_3_].

The infrared spectra are shown in Figure 2. The primary spectral data are listed in Table 2.

The IR spectra of the ligand and chromium complex are shown in Figure 1 and Table 2. The carbonyl stretching vibration frequency of the 4-position of BCA was located at 1653 cm^−1^, forming a complex that moved to 1627 cm^−1^. A shift of 26 cm^−1^ in the direction of a low wavenumber indicated that the 4-carbonyl oxygen of the ligand participated in the coordination. The stretching vibration ν (C = C) of the benzene ring in which 5-OH existed was obviously weakened in the complex, and the wavenumber also decreased. The stretching vibration frequency (3388 cm^−1^) of the hydroxyl group of BCA increased by 44 cm^−1^ after the formation of the complex, which was caused by the coordination of 5-OH. The stretching vibration of the aromatic ether bond ν (C-O-C) in the ligand had no changes with the formation of the complex, indicating that the oxygen atom on the aromatic ether bond of BCA did not participate in the coordination.

#### 2.1.4. UV Spectrum

**Figure 3:**
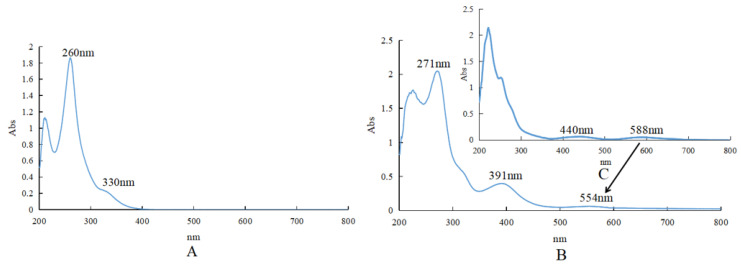
UV spectra. (**A**) UV spectra of BCA, in the solvent of CH3OH, [BCA]=5 × 10^−5^ mol·L^−1^. (**B**) UV spectra of [CrBCA3], in the solvent of CH3OH, [CrBCA3] = 5 × 10^−5^ mol·L^−1^. (**C**) UV spectra of Cr(CH3COO)3, in the solvent of CH3OH, [Cr(CH3COO)3] = 1 × 10^−4^ mol·L^−1^.

As shown in Figure 3, BCA had two characteristic UV absorption peaks at 260 and 330 nm (weak) in the solvent of CH_3_OH. After the formation of the complex, the two peaks shifted to the long-wavelength direction, and the maximum absorption wavelengths were 271 and 391 nm, respectively, in the solvent of CH_3_OH. The strong absorption band II shifted from 260 nm to 271 nm, and the weak absorption band I also showed a large displacement (from 330 nm to 391 nm). For BCA, the B ring could not be conjugated with the unsaturated carbonyl group of the C ring, so the absorption intensity of band I decreased to acromion. However, in the complex, the coordination of three BCA molecules with chromium through the 4-carbonyl group and 5-hydroxyl group enhanced the planar structure of the whole molecule. The increase in the conjugated system led to the red shift of band I and band II, and a slight increase in the strength of band I and band II. Cr(III) of Cr(CH_3_COO)_3_ had d–d transition absorption peaks at 440 nm [*ν*_2_ (^4^*A*_2g_→^4^*T*_1g_ (F))] and 588 nm [*ν*_1_ (^4^*A*_2g_→^4^*T*_2g_)]. After the formation of the complex, the d–d transition absorption peak at 588 nm shifted to 554 nm. The other d–d transition absorption peak was covered by the BCA absorption peak. UV data also showed that 4-carbonyl and 5-hydroxyl of BCA were involved in the coordination.

#### 2.1.5. X-ray Powder Diffraction

**Figure 4:**
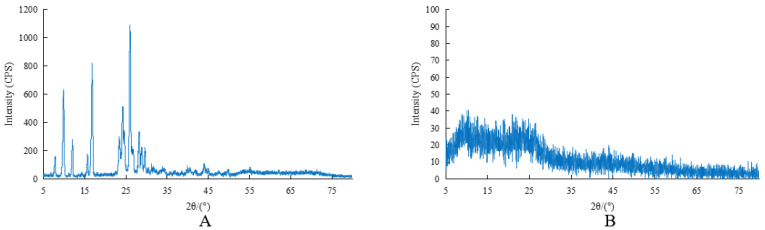
X-ray powder diffraction patterns. (**A**) X-ray powder diffraction pattern of BCA. (**B**) X-ray powder diffraction pattern of [CrBCA_3_].

As shown in Figure 4, the X-ray powder diffraction patterns of BCA and [CrBCA_3_] were very different. BCA had a good crystal morphology. After the complex of BCA and Cr was formed, it could not easily form a good crystal morphology and become an amorphous compound.

**Figure 5:**
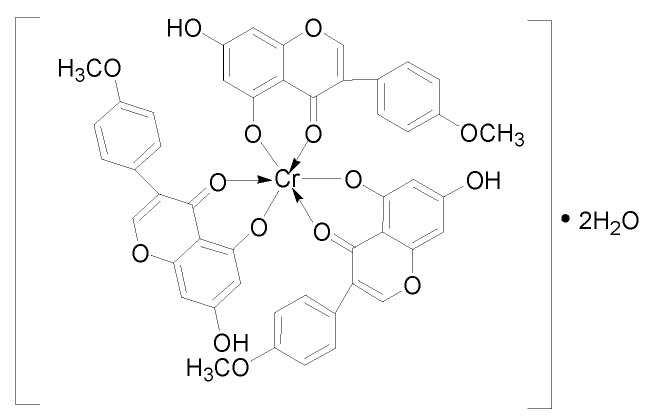
The structure of [CrBCA_3_].

On the basis of the above results, the structure of the chromium complex of BCA could be inferred as shown in Figure 5. The chemical formula was inferred as CrC_48_H_33_O_15_•2H_2_O. After chelation, the chromium content of [CrBCA_3_] was 55 μg/mg.

### 2.2. Biochemical Results

#### 2.2.1. Animal Growth

**Figure 6:**
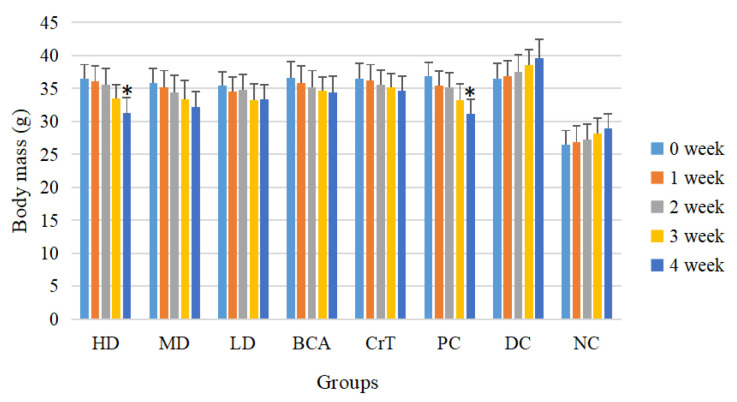
Comparison of body mass changes. Values are means ± SD (*n* = 7). There were significant differences in body weight before and after the experiment (denoted by “*” for *p* < 0.05).

Figure 6 shows the weight comparison for every week before and after the experiment in each group of mice.

As shown in Figure 6, db/db mice were heavier, and the HD mice lost the most weight after treatment in all three test groups. The change in body mass in the HD group was statistically significant compared with that before treatment (*p* < 0.05). The body mass of the BCA group also decreased, but there was no significant difference compared with that before treatment (*p* > 0.05). The CrT group showed some weight loss, but there was no significant difference compared with that before treatment (*p* > 0.05). The activity of weight loss was also better in the PC group, and the difference was statistically significant compared with that before treatment (*p* < 0.05). The DC group continued to gain weight. The body mass of mice in the NC group showed a normal increase. The weight loss in the HD group was similar to that in the PC group.

#### 2.2.2. Determination of Fasting Blood Glucose

Table 3 shows the changes in fasting blood glucose in each group during the experiment. The HD group showed the best hypoglycemic activity among the three test groups. There were significant differences in blood glucose between the HD group and DC group in the second, third and fourth weeks (*p* < 0.01). The PC group also showed good hypoglycemic activity, and there was no significant difference between the HD group and PC group. This showed that [CrBCA_3_] had good hypoglycemic activity. The BCA group demonstrated certain hypoglycemic activity, but its hypoglycemic activity was not as good as that of the HD group (*p* < 0.05 or *p* < 0.01). The results showed that chromium played an important role in reducing blood sugar. The CrT group had hypoglycemic activity, but its hypoglycemic activity was not as good as that of the HD group (*p* < 0.05).

#### 2.2.3. Determination of Random Blood Glucose

Table 4 shows the changes in random blood glucose values in each group over the course of 4 weeks. The hypoglycemic effect of the HD group was better than that of the MD group and LD group (*p* < 0.05 or *p* < 0.01). The BCA group exhibited a certain hypoglycemic effect, but it was not as effective as the HD group (*p* < 0.05 or *p* < 0.01). The CrT group had hypoglycemic activity, but its hypoglycemic activity was not as good as that of the HD group (*p* < 0.05). These results showed that the complex formed by chromium and BCA played a synergistic role in the hypoglycemic effect.

#### 2.2.4. Determination of Liver/Body and Kidney/Body Ratios

**Figure 7:**
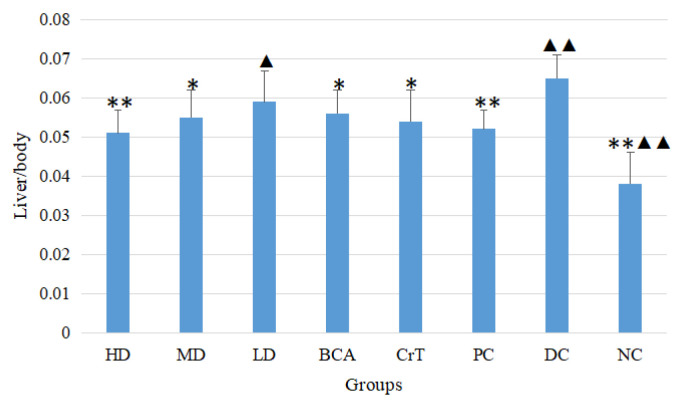
The liver/body ratio after 4 weeks. Values are means ± SD (*n* = 7). The differences between these groups and the DC group were statistically significant (denoted by “*” for *p* < 0.05, “**” for *p* < 0.01); the differences between these groups and the PC group were statistically significant (denoted by “^▲^” for *p* < 0.05, “^▲▲^” for *p* < 0.01).

**Figure 8:**
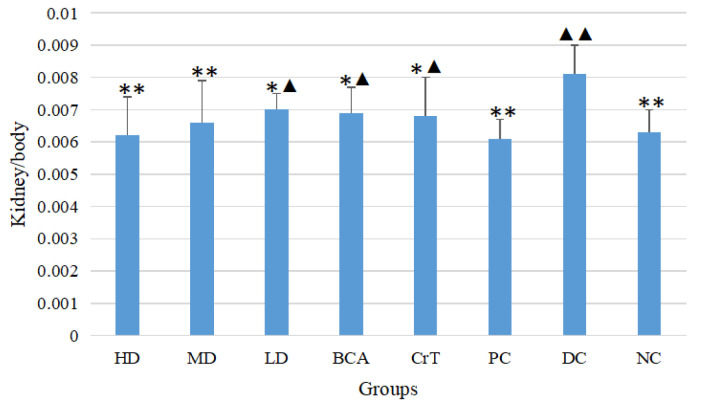
The kidney/body ratio after 4 weeks. Values are means ± SD (*n* = 7). The differences between these groups and the DC group were statistically significant (denoted by “*” for *p* < 0.05, “**” for *p* < 0.01); the differences between these groups and the PC group were statistically significant (denoted by “▲” for *p* < 0.05, “^▲▲^” for *p* < 0.01).

The liver-to-body ratios of each group after 4 weeks are shown in Figure 7. Compared with the DC group, the HD and MD groups had significantly improved liver-to-body ratios (*p* < 0.05 or *p* < 0.01). The HD group had the best effect among the three test groups, and there was no significant difference between the HD group and PC group (*p* > 0.05). The BCA group also showed some improvement in the liver-to-body ratio, but its activity was not as good as the HD group. The CrT group showed improvement in the liver-to-body ratio, but its activity was not as good as the HD group. The kidney-to-body ratios of each group after 4 weeks are shown in Figure 8. Compared with the DC group, the HD, MD and LD groups had significantly improved kidney-to-body ratios (*p* < 0.05 or *p* < 0.01). The HD group had the best effect among the three test groups. The BCA group also showed some improvement in the kidney-to-body ratio, but its activity was not as good as that of the HD group. The CrT group showed an improvement in the kidney-to-body ratio, but its activity was not as good as the HD group. These results suggested that [CrBCA_3_] had the ability to repair tissue damage caused by hyperglycemia.

#### 2.2.5. Comparison of OGTT

**Figure 9:**
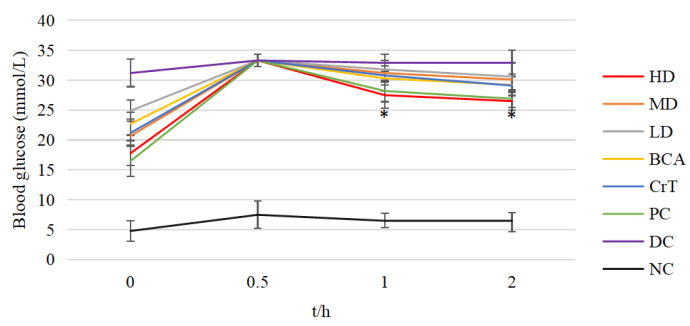
OGTT in each group of mice. Values are means ± SD (*n* = 7). The differences between these groups and the DC group were statistically significant (denoted by “*” for *p* < 0.05).

After 4 weeks, the OGTT results for each group were obtained (Figure 9). The glucose tolerances of the HD group and PC group were better than that of the DC group after 1 h of glucose administration (*p* < 0.05). The glucose tolerances of the HD, BCA, CrT and PC groups were better than that of the DC group after 2 h of glucose administration (*p* < 0.05). This indicated that [CrBCA_3_] could improve the glucose tolerance of db/db mice.

#### 2.2.6. Comparison of ITT

**Figure 10:**
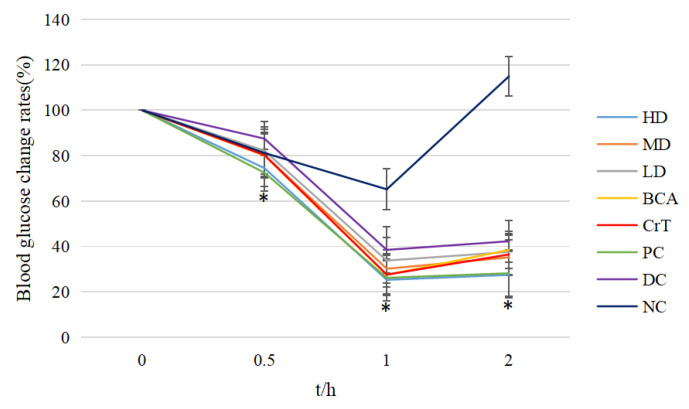
ITT in each group of mice. Values are means ± SD (*n* = 7). The differences between these groups and the DC group were statistically significant (denoted by “*” for *p* < 0.05).

Figure 10 shows the results of ITT in each group after 4 weeks of administration. The results of ITT in the HD and PC groups were better than that in the DC group after 0.5 h of insulin administration (*p* < 0.05). The results of ITT in the HD, MD, BCA, CrT and PC groups were better than that in the DC group after 1 h of insulin administration (*p* < 0.05). The results of ITT in the HD and PC groups were better than that in the DC group after 2 h of insulin administration (*p* < 0.05). These findings suggested that [CrBCA_3_] had similar activity to a positive drug in increasing insulin sensitivity, and its effect was better than in the BCA and CrT groups.

#### 2.2.7. Histopathological Observation

##### Morphological Changes in Liver Tissue

**Figure 11:**
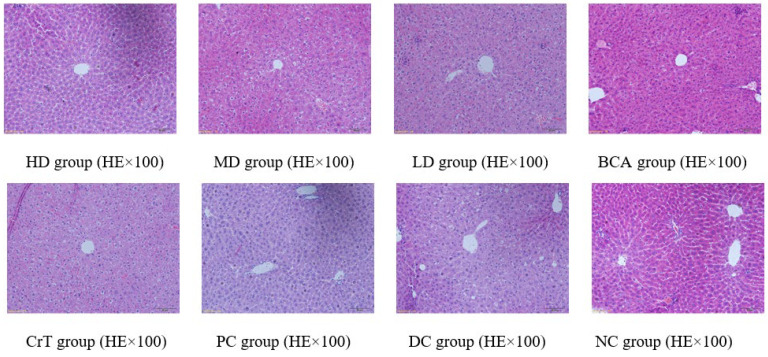
Morphological changes in liver tissue.

After HE staining, the morphology of the liver was observed under a microscope, as shown in Figure 11.

Figure 11 shows the pathological changes in the liver. NC group: The liver structure in mice was complete and clear. The hepatic plates and hepatic sinusoids were arranged radially around the central vein. No abnormal changes were noted in the liver sinus, and the structure was clear. Liver cells were polygonal, normal in shape, and the cytoplasm was distributed evenly. HD group and PC group: Liver cells were clear, with mild hepatic fibrosis, and the fatty degeneration of the liver was mild. MD group, BCA group and CrT group: The fatty degeneration of the liver was mild. The liver cells were clear and complete. The nuclear and cytoplasmic separation showed a significant improvement. The arrangement of cells was regular and the form of cells was complete. LD group: Hepatic cells developed serious fatty degeneration and exhibited cavitation. The nuclear and cytoplasmic separation was obviously serious. Partial hepatic cells were apoptotic. DC group: The boundaries of hepatic lobules in mice were relatively unclear. The structure of the liver sinus was blurred. The liver cells exhibited fatty degeneration and a disordered arrangement. The interspace in nuclear membranes was separated and the cytoplasm dissolved. Hepatic fibrosis was serious.

##### Morphological Changes in Renal Tissue

**Figure 12:**
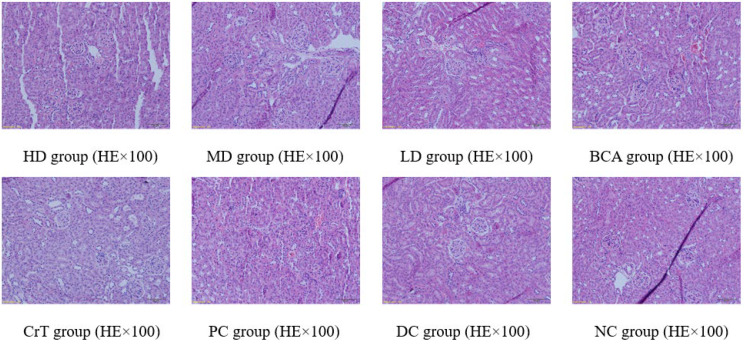
Morphological changes in renal tissue.

After HE staining, the morphology of the kidney was observed under a microscope, as shown in Figure 12.

Figure 12 shows the pathological changes in the kidney. NC group: The glomerular volume was normal, and the capillaries were clearly visible. HD group and PC group: The glomerular volume was normal, and the renal tubules were slightly narrow. MD group, BCA group and CrT group: The glomerular volume was normal, and the kidney tubule interstitial spaces showed slight fibrosis. LD group: The glomerulus showed proliferation and hypertrophy. The glomerular basement membrane was hyperplastic. Partial renal tubular epithelial cells appeared granulovacuolar degeneration. DC group: The glomerular volume was increased and renal vesicles were fissured. The glomerular basement membrane was hyperplastic. Renal tubular epithelial cells displayed granulovacuolar degeneration.

##### Morphological Changes in Pancreatic Tissue

**Figure 13:**
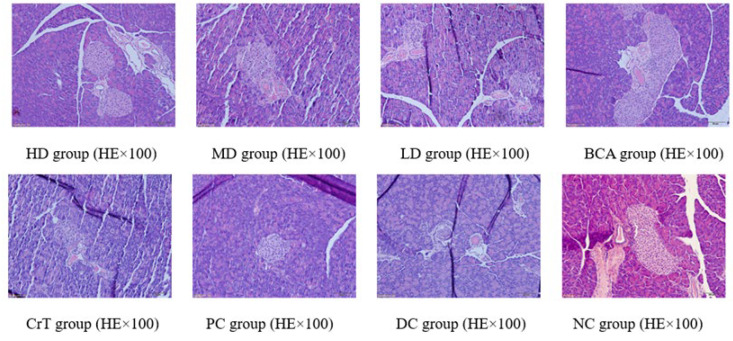
Morphological changes in pancreatic tissue.

After HE staining, the morphology of the pancreas was observed under a microscope, as shown in Figure 13.

Figure 13 shows the pathological changes in the pancreas. NC group: The boundary between the pancreatic islet and exocrine glands was clear, and the pancreatic islet form was regular. The number of cells in the pancreatic islet was large and closely arranged, and the cells were distributed evenly. HD group, MD group and PC group: The degree of degranulation of pancreatic islet mast cells was significantly reduced. The fibrosis of the pancreatic islet was reduced. Heterotopic deposition of fat cells was rare. Mast cells showed a reduction. LD group, BCA group and CrT group: The degree of degranulation of pancreatic islet mast cells was heavy, but slightly lighter than that of the model group. Partial pancreatic islets showed fibrosis, and mast cells appeared. DC group: The pancreatic islet mast cells presented granulovacuolar degeneration. Pancreatic islet fibrosis was observed, and many collagenous fibers were deposited in the pancreatic islet. The fat cells exhibited heterotopic deposition. There was lymphocyte infiltration in the pancreas. Mast cells increased.

##### Morphological Changes in Skeletal Muscle Tissue

**Figure 14:**
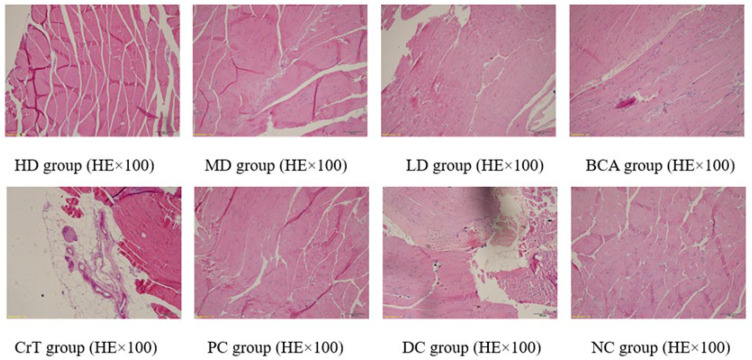
Morphological changes in skeletal muscle tissue.

After HE staining, the morphology of the skeletal muscle was observed under a microscope, as shown in Figure 14.

Figure 14 shows the pathological changes in skeletal muscle. NC group: The muscle tissue was arranged completely, regularly and clearly. The muscle cells were uniform in size, with no atrophy, oedema, necrosis or rupture of muscle cells, or proliferation of fibrous tissue. HD group, MD group and PC group: The muscle tissues were arranged regularly, and the muscle cells were even in size. Scattered foci of myocyte atrophy were observed. Myocyte edema was small in area and mild in degree. Mild inflammatory cell infiltration and muscle cell necrosis and rupture were rare. LD group, BCA group and CrT group: The arrangement of muscle tissue was disordered and the muscle cells were not uniform in size. Muscle cells showed mild atrophy. The area of muscle cell edema was large. Inflammatory cell infiltration was mild, and the area of focal muscle tissue rupture was large. No obvious fibrosis was observed. DC group: The arrangement of muscle tissue was disordered. The myocyte volume was reduced, and the atrophy was obvious. The area of muscle cell edema was large. Inflammatory cell infiltration was more obvious. The area of focal muscle tissue rupture was larger. No obvious fibrosis was observed.

#### 2.2.8. Determination of Serum Biochemical Indices

Table 5 shows the levels of lipid indicators in the serum of normal and diabetic mice.

As shown in Table 5, for TC, the PC group and HD group showed a noticeable improvement, and there was no significant difference compared with the NC group (*p* > 0.05). For TG, the PC group and HD group were significantly lower than the DC group (*p* < 0.05). After administration, the HDL-C of the PC group and HD group significantly increased compared with the DC group, and the difference was statistically significant (*p* < 0.01). For LDL-C, in the PC group and HD group, compared with the DC group, there was a significant decrease, and the difference was statistically significant (*p* < 0.05). For GSP, the PC group and HD group showed improvements, and compared with the NC group, there was no significant difference (*p* > 0.05). Compared with the DC group, NEFA in the PC group and HD group decreased significantly (*p* < 0.05). The BCA group and CrT group showed some improvement in serum biochemical indicators, but its activity was not as good as that of [CrBCA_3_]. Thus, BCA formed a complex with chromium, which could play a better role in the regulation of serum biochemical indicators.

#### 2.2.9. Effects on MDA Levels and Antioxidant Enzyme Activities in the Liver

As shown in Table 6, compared with the DC group, MDA in the PC group and HD group was significantly improved (*p* < 0.01). Compared with the DC group, CAT in the HD group and MD group was significantly improved (*p* < 0.01). For SOD, the PC group and HD group showed a significant improvement, and the differences were statistically significant compared with the DC group (*p* < 0.01). For GSH-Px, there was no significant difference between the HD group and NC group (*p* > 0.05). Compared with the DC group, the MD group and LD group showed a significant improvement in GSH-Px (*p* < 0.01 or *p* < 0.05). The BCA group and CrT group showed improvements in the related indices to some extent, but the effect was not as good as that of the complex. This showed that the coordination between BCA and Cr(III) exerted a better effect in improving the antioxidant capacity.

#### 2.2.10. The Effects on Glycogen Levels of db/db Mice

**Figure 15:**
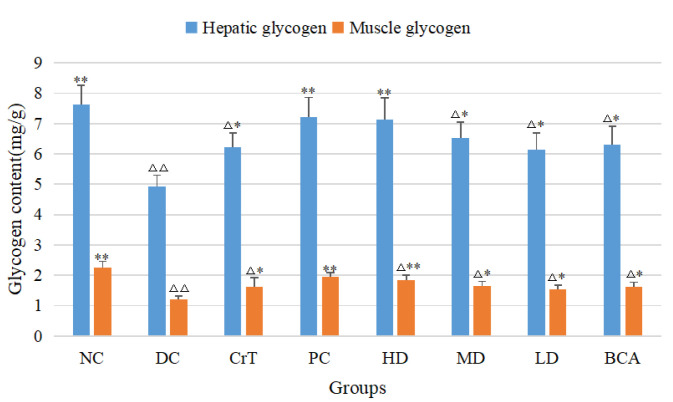
Effects of [CrBCA_3_] on glycogen levels in db/db mice. Values are means ± SD (*n* = 7). The differences between these groups and the NC group were statistically significant (denoted by “^△^” or “^△△^” for *p* < 0.05 or *p* < 0.01, respectively); the differences between these groups and the DC group were statistically significant (denoted by “^*^” or “**” for *p* < 0.05 or *p* < 0.01, respectively).

As shown in Figure 15, for hepatic glycogen, the HD group had a significant improvement; compared with the NC group, there was no significant difference (*p* > 0.05). Compared with the DC group, all of the complex groups were significantly improved (*p* < 0.05 or *p* < 0.01). As for muscle glycogen, there was a significant difference between the groups of the complex and the DC group (*p* < 0.05 or *p* < 0.01), and the complex had a good effect in promoting muscle glycogen. The PC group and HD group had almost the same effect in improving the content of glycogen. The BCA group and CrT group had better effects in enhancing hepatic glycogen and muscle glycogen compared with the DC group (*p* < 0.05), but the effects were not as good as the complex.

### 2.3. Sub-Acute Toxicity Study in Mice

**Figure 16:**
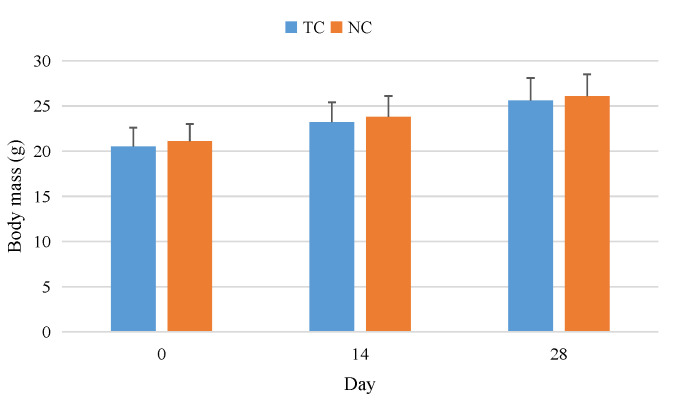
Effects of [CrBCA_3_] on the body mass of the mice in sub-acute toxicity study. Values are means ± SD (*n* = 10).

**Figure 17:**
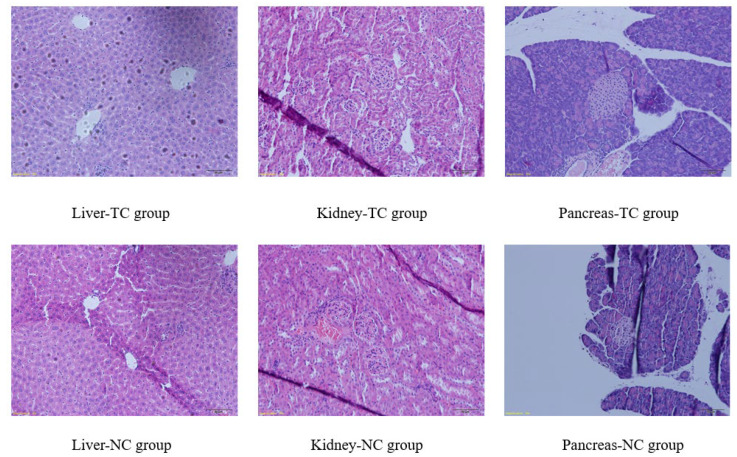
Effects of [CrBCA_3_] on some microanatomical features of the rats in sub-acute toxicity studies.

After the sub-acute toxicity study, no mice (either male or female) died. The results showed that there were no abnormalities in the behavior, eating, drinking, appearance, feces, urine, secretion and mental state of mice. As shown in Figure 16, after one month of the sub-acute toxicity study, there was no significant difference in body weight between the TC group and NC group, indicating that the complex had no effect on the digestive systems of mice. As shown in Table 7, there was no significant difference in organ index between the TC group and NC group. Figure 17 showed that the complex also did not cause significant pathological damage to the liver, kidney or pancreas. Table 8 shows that the random blood glucose and insulin levels of the two groups of mice were comparable and within the normal ranges. There were no significant differences in TC, TG, LDL-C, HDL-C, GSP and NEFA levels. MDA, CAT, SOD and GSH-Px in the TC group were not significantly different from those in the NC group. The results showed that the complex had no significant effect on glucose metabolism, lipid metabolism and antioxidant capacity in mice. As shown in Table 9, a blood routine test showed no significant differences in WBC, RBC, HGB and PLT between the TC group and NC group. As shown in Table 9, blood biochemical indices showed no significant differences in liver function and kidney function between the TC group and NC group. The sub-acute toxicity test showed that the complex had no sub-acute toxicity to normal mice.

## 3. Materials and Methods

### 3.1. Chemicals and Instruments [42]

Biochanin A (Ci Yuan Biotechnology Co., Ltd. Shanxi, China, batch number 20190721, purity > 99%). CrCl_3_•6H_2_O (Shanghai ZZBIO Co., Ltd., Shanghai, China, purity > 99%). (CH_3_COO)_3_Cr (Shanghai MackLin Biochemical Technology Co., Ltd., Shanghai, China, purity > 99%). Glibenclamide was purchased from Sigma-Aldrich (Milwaukee, WI, USA). Total cholesterol (TC), triglyceride (TG), low-density lipoprotein cholesterol (LDL-C), high-density lipoprotein cholesterol (HDL-C), glycosylated serum protein (GSP), nonesterified fatty acids (NEFA), malondialdehyde (MDA), catalase (CAT), superoxide dismutase (SOD), glutathione peroxidase (GSH-Px), alanine transaminase (ALT), aspartic transaminase (AST), alkaline phosphatase (ALP), total protein (TP), albumin (ALB), blood urea nitrogen (BUN), creatinine (CR) and glycogen test kits were obtained from Jiancheng Bioengineering Institute (Nanjing, China). Glucocard glucometer (ARKRAY, Inc., Kyoto, Japan), Arkray blood sugar test paper (ARKRAY, Inc., Kyoto, Japan), Biosynthetic Human Insulin Injection (Novolin R, Novo Nordisk China). All chemicals and solvents used were of analytical or high-performance liquid chromatography grade. The elemental analyses were determined on a Vario EL III analysis elementary analyzer (Elementar Instruments Co., Hanau, Germany). UV spectra (200–800 nm) were recorded on a TU-1810PC UV–visible spectrophotometer (Persee Instruments Co., Beijing, China). Infrared spectra of powdered samples (KBr disks) were recorded on a Nexus470 Fourier transform infrared spectrometer (Thermo Nicolet Co., Waltham, MA, USA) from 4000 to 400 cm^−1^. The chromium content was determined by atomic absorption spectroscopy with a AA-3800G atomic absorption spectrometer (Shanghai Metash Instruments Co., Ltd., Shanghai, China). Thermodynamic analysis was performed using a SLTG DTA-TG thermal analyzer (Sangli Electronic Instruments Co., Ltd., Nanjing, China). High-resolution mass spectra (HR-MS) were obtained on a SCIEX X-500R QTOF (ESI mode, SCIEX Co., Framingham, MA, USA). X-ray powder diffractometer (D8 ADVANCE, Bruker Corporation, Karlsruhe, Germany). Coulter JT automatic blood cell analyzer (Beckman Coulter, Inc., Brea, CA, USA). 7150 automatic biochemical analyzer (Hitachi, Ltd., Tokyo, Japan). HC-2518 High-Speed Centrifuge (Anhui Zhongke Zhongjia Science Instrument Co., Ltd., Hefei, China).

### 3.2. Experimental Animals [42]

db/db mice (BKS.Cg-m +/+ Leprdb, 35 ± 3 g) + C57 (BKS, 25 ± 2 g) mice were selected from the Model Animal Research Center of Nanjing University (Certificate number: SCXK (Su) 2015-0001). Kunming mice (20 ± 2 g) were obtained from the institute of Beijing Vital River Laboratory Animal Technology Co., Ltd. (Certificate number: SCXK (Jing) 2016-0006). All the animals were kept and maintained under 21 ± 1 °C, at humidity of 60 ± 5%. All utensils and food used in mice were sterilized. Mice were free to eat food and drink distilled water. The padding was kept dry, and the mice were subjected to 12 h of alternating light.

These experimental procedures were in compliance with the regulations of the National Institutes of Health (NIH) of the USA and conducted in accordance with the guidance of the Animal Ethics Committee of Beijing University of Chinese Medicine and the ARRIVE guidelines and regulations. The study protocols were approved by the Animal Ethics Committee of Beijing University of Chinese Medicine (No. BUCM-4-2021031709-5011).

### 3.3. Synthesis and Structural Characterisation of [CrBCA_3_]

Biochanin A (1.42 g, 5.00 mmol) was added to 50 mL anhydrous ethanol and heated at 60 °C for 0.5 h. Once the biochanin A was completely dissolved, we added (CH_3_COO)_3_Cr (0.68 g, 3.00 mmol). The mixture was stirred under reflux for 18 h. The mixture was then cooled to room temperature. A green precipitate formed and was filtered. After washing each sample three times with deionized water (50 mL each time) and anhydrous ethanol (50 mL each time), the precipitate was collected and dried in a vacuum to obtain a grass-green powder solid with a yield of 51.2%.

The structure of [CrBCA_3_] was determined by elemental analysis, atomic absorption spectroscopy, LC-MS, thermal analysis (under the condition of 10 °C/min heating rate in flowing air), infrared spectroscopy and UV–visible spectroscopy (200–800 nm). The crystal morphology of BCA and [CrBCA_3_] was analyzed on an X-ray powder diffractometer. The X-ray powder diffractometer was operated at 40 kV and 40 mA, produced by Cu Kα radiation.

### 3.4. Animal Hypoglycemic Experiment Design

Forty-nine db/db mice were randomly divided into seven groups (each group comprised three males and four females). Among them, three groups of db/db mice were test groups. In combination with studies, the maximum daily dose of chromium in the human body is 1 mg, i.e., 1 mg/65 kg = 0.015 mg/kg [43]. The equivalent dose for mice is 9.01 times that for humans. The animal equivalent dose was 9.01 × 0.015 mg/kg = 0.135 mg/kg (135 μg Cr/kg). Therefore, three test groups of db/db mice were given low (0.45 mg/kg per day, LD), medium (0.90 mg/kg per day, MD) and high (1.80 mg/kg per day, HD) doses of [CrBCA_3_] (equivalent of 25, 50 and 100 μg Cr/kg body weight, respectively, all less than 135 μg Cr/kg) by daily oral gavage, respectively. In the BCA group (BCA), all the mice (*n* = 7) were treated with 1.64 mg BCA/kg body weight (because there were 3 ligands in one complex molecule, the dose in the BCA group was 3 times the molar amount in the high-dose group). The negative control group was the CrCl_3_•6H_2_O-treated group (CrT), and all the mice (*n* = 7) were treated with 0.51 mg CrCl_3_•6H_2_O/kg body weight (equivalent of 100 μg Cr/kg body weight) by daily oral gavage. In the positive control group (PC), all the mice (*n* = 7) were treated with 1.00 mg glibenclamide/kg body weight by daily oral gavage (the dosage was 9.01 times the usual dose of glibenclamide (5–10 mg/day, taking 7.2 mg/day), so the dose of PC group is (7.2 mg ÷ 65 kg) × 9.01 = 1 mg/kg). In the diabetic control group (DC), all the mice (*n* = 7) were treated with 0.9% saline by daily oral gavage. C57 mice (*n* = 7), as the normal control group (NC), were treated with 0.9% saline by daily oral gavage. The preparation method of the oral administration solution of each group of mice was as follows: each group of compounds was mixed with distilled water to prepare suspensions, which were prepared fresh before each use. The filling volume was 0.1 mL/10 g. All the above eight groups of mice had sustained oral gavage at around 10 a.m. once daily for 4 weeks.

### 3.5. Biochemical Assays [42]

Body mass and blood glucose measurements: The animals’ weights were determined and recorded weekly. The tail vein blood of mice was collected and the blood glucose level was measured by a glucometer. Random blood glucose levels were measured at 8 a.m. every Friday. Fasting blood glucose levels were measured at 8 a.m. every Saturday.

OGTT [44]: At the last week of treatment, the mice were given 2 g glucose/kg oral glucose (40% aqueous solution) after fasting for 12 h. The blood glucose values were measured at 0 min (before administration) and 30, 60 and 120 min after administration. ITT [45]: At the last week of treatment, after fasting for 4 h, 1 U/kg of Novolin was injected intraperitoneally, and the glucose values were measured at 0 min (before injection) and 30, 60 and 120 min after injection. At 0 min, the blood glucose level was 100%, and the blood glucose levels were calculated at each time point.

At the end of the experiment, all mice were fasted overnight with free access to water. Blood samples were collected from each group of mice by post-orbital puncture. The serum was separated from blood by centrifugation at 3000× *g* for 10 min at 4 °C. Serum biochemical parameters (TC, TG, HDL-C, HDL-C, GSP and NEFA) were determined according to the instructions of the test kit. Antioxidant enzyme activities of the liver were determined according to the literature [46]. In brief, liver tissue was homogenized with Tris–HCl (5 mmol/L, pH 7.4). The supernatant was obtained by centrifugation (1000× *g*, 15 min, 4 °C). The CAT, SOD and GSH-Px activities and the MDA content were measured according to the instructions of the test kit. Glycogen levels in the liver and skeletal muscle were measured using the test kit.

On the last day of the experimental period, all the experimental mice were euthanized by CO_2_ suffocation. The liver, kidney, pancreas and skeletal muscle of the mice were collected and washed with ice-cold 0.9% NaCl solution, and then drained by filter paper. Liver weight and left kidney weight were measured accurately, and the organ index was calculated. Some of the samples were fixed in 10% neutral buffered formalin for 24 h for further histopathological examination. The liver, kidney, pancreas and skeletal muscle of mice stained with hematoxylin and eosin (H&E) were observed under a microscope.

### 3.6. Sub-Acute Toxicity Test [42]

We used OECD Test No. 407: Repeated Dose 28-Day Oral Toxicity Study in Rodents. The sub-acute toxicity test of the complex in KM mice was carried out by the maximum limit method. Twenty healthy mice were randomly divided into the negative control group and experimental group (10 mice in each group, 5 males and 5 females). The toxicity control group (TC) was treated with the complex at a dose of 1.5 g/kg/day by oral gavage (LD_50_ was not detected, so the dose for sub-acute toxicity test was 1/10 of the maximum dose of 15 g/kg, which had been measured before). The normal control group (NC) was given 0.2 mL/day normal saline orally. Throughout the experiment, the mice were given a normal diet. The animals were observed at least twice a day and their mental state, behavior and coat color were recorded. Body weight was measured on days 0, 14 and 28, respectively. All the mice were euthanized after four weeks of administration of [CrBCA_3_], and execution was the same as in Section 3.5. The lipid profiles and antioxidant enzyme activities of mice were measured and compared with those of normal mice. The liver, pancreas, kidneys, heart, thymus and spleen were collected and weighed. The liver, pancreas and kidneys were immobilized with 10% formalin. These organs were sliced and stained with H&E for histopathological observation under an electron microscope.

### 3.7. Statistical Analysis

The data were given as means ± SEM. Comparison between experimental groups were made by using one-way ANOVA, followed by Student–Newman–Keuls test. *p* values < 0.05 or < 0.01 were considered significant difference or extremely distinct differences (SPSS(PASW) 20.0).

## 4. Discussion

In this study, a novel chromium(III) complex of BCA was synthesized. After elemental analysis, mass spectrum, TG-DTA, infrared spectrum, UV–visible spectrum analysis and X-ray powder diffraction studies, it was found that the coordination geometry was composed of three BCA ligands and chromium(III). The binding sites of the ligands were 5-hydroxy and 4-carbonyl, and the complex contained two molecules of water, which were lost at 85 °C.

In the hypoglycemic activity research, the db/db mice were selected as the hypoglycemic activity model. The pathogenesis of db/db mice was similar to that of human type 2 diabetes, such as polydipsia, polyphagia, polyuria, obesity, hyperglycemia, hyperinsulinemia, insulin resistance, abnormal lipid metabolism and so on [47,48]. [CrBCA_3_] was found to reduce body weight in db/db mice and to improve both fasting and random blood glucose levels. The liver-to-body ratio and kidney-to-body ratio of db/db mice were also significantly improved, indicating that the complex had a good repair effect regarding tissue injury. OGTT and ITT showed that the complex significantly improved glucose and insulin tolerance in db/db mice.

Histopathology showed that the complex could repair various organ injuries (liver, kidney, pancreas and skeletal muscle) caused by diabetes. The pathological injury of each organ had recovered. The results showed that the complex could repair the related organ damage caused by diabetes, and improve the symptoms of diabetes. Thus, the complex was beneficial for treating diabetes at its source.

Type 2 diabetes is associated with disorder of lipid metabolism. Krol et al. [49] and Sharma et al. [50] reported that chromium supplementation significantly reduced serum total cholesterol, low-density lipoprotein and triglyceride levels in type 2 diabetic rats. Sahin et al. [51] reported that chromium picolinate supplementation significantly reduced serum total cholesterol and triglyceride levels in patients with type 2 diabetes. In this study, the complex significantly reduced serum TC, LDL-C and TG, and increased HDL-C, in db/db mice. In addition, the levels of NEFA and GSP were also decreased.

Hyperglycemia and oxidative stress formed a vicious cycle, leading to the destruction of islet cells and insulin resistance, which eventually led to the occurrence and development of diabetes and its complications [52,53]. Many studies had shown that antioxidant therapy could delay the onset of diabetes and its complications [54,55,56]. In this study, the content of MDA was decreased, and the content of CAT, SOD and GSH-Px was increased. The results showed that the complex could enhance the antioxidant capacity of the db/db mice and improve the oxidative stress injury caused by hyperglycemia.

Glycogen is stored primarily in the liver and skeletal muscle, and type 2 diabetes is associated with decreased glycogen concentrations. In this study, the concentrations of liver and muscle glycogen were increased. The results showed that the complex could promote the synthesis of glycogen in vivo.

At present, the toxicological reports on organic chromium compounds are not quite in agreement, and it has been found that organic chromium compounds are not toxic when used in a certain range, and they did not cause toxicity to the body under physiological conditions [7,57]. Other studies found that organic chromium compounds had cytotoxicity and genotoxicity [58], and their safety has attracted widespread attention. In this study, the sub-acute toxicity study showed that the high-dose complex did not result in death, did not cause abnormal changes in spirit, behavior and fur and did not lead to significant changes in body weight. There was no significant difference in organ indices compared with normal mice. The pathological sections of the liver, kidney and pancreas were not significantly different from those of normal mice. There was no abnormality in the indices of serum and antioxidant capacity of mice. There were also no significant differences in blood routine tests and liver and kidney function between the TC group and NC group. The results showed that the complex did not produce sub-acute toxicity in normal mice.

Our other studies showed that the ligand had a positive effect on the bioavailability of chromium. We will also use some compounds with good hypoglycemic activity and high oral bioavailability as ligands in the study of chromium complexes, such as iminosugar, which is a good choice [59,60,61].

## 5. Conclusions

In this study, the new complex of chromium(III) with BCA was synthesized and its chemical formula was [CrBCA_3_]·2H_2_O. The study of its hypoglycemic activity showed that it could lower the weight of db/db mice and lower the fasting blood glucose and random blood glucose and had a good hypoglycemic effect. Liver-to-body ratio and kidney-to-body ratio were also improved. For the liver, kidney, pancreas and skeletal muscle injury caused by diabetes, it showed good repair activity. Glycogen synthesis was improved, and the degree of insulin resistance was reduced. The serum lipid level and antioxidation ability of the body were significantly improved, which were beneficial for recovery from diabetes. The sub-acute toxicity study showed that the complex had no potential toxicity and did not cause abnormal changes in normal mice. [CrBCA_3_] had good hypoglycemic activity, the ligand had no obvious toxicity and the complex had good safety. This study expands the research scope of hypoglycemic active chromium complexes, and provides a reference for the research and development of new foods and drugs containing chromium.

## Figures and Tables

**Table 1:** Elemental and MS analysis of the complex.

C%		H%		Cr%		Molecular Weight	
test	theory	test	theory	test	theory	test	theory [M-2H_2_O-H]^−^
61.43	61.47	3.93	3.95	5.53	5.55	899.8812	900.1146

**Table 2:** The primary IR spectral data of biochanin A and the chromium complex (cm^−1^).

Compound	ν(O-H)	ν(C = O)	ν(C = C)	ν(C-O-C)
Biochanin A	3388	1653	1623	1249
[CrBCA_3_]	3432	1627	1612	1249

**Table 3:** Changes in fasting blood glucose level.

Groups	*n*	Fasting Blood Glucose (mmol/L)				
		Week 0	Week 1	Week 2	Week 3	Week 4
HD	7	30.1 ± 1.3	28.2 ± 2.3	24.1 ± 2.6 **	20.2 ± 3.2 **	17.4 ± 2.1 **
MD	7	30.2 ± 1.2	29.5 ± 3.0	26.1 ± 2.3 *	23.3 ± 2.1 **^,^^★^	20.2 ± 2.4 **^,^^★^
LD	7	31.3 ± 2.1	30.5 ± 2.3	28.2 ± 2.1 *^,^^★^	26.1 ± 2.3 *^,^^★★^	24.3 ± 2.1 **^,^^★★^
BCA	7	30.3 ± 2.2	28.2 ± 2.1	26.6 ± 2.3 *	24.6 ± 2.2 **^,^^★^	22.4 ± 1.8 **^,^^★★^
CrT	7	30.1 ± 2.1	29.2 ± 2.9	26.5 ± 3.1 *	23.1 ± 2.3 **^,^^★^	21.5 ± 2.5 **^,^^★^
PC	7	31.4 ± 2.3	28.9 ± 3.1	24.7 ± 2.3 **	20.5 ± 2.1 **	16.2 ± 2.2 **
DC	7	30.3 ± 2.1	30.5 ± 2.4	31.5 ± 3.2 ^★★^	31.8 ± 2.1 ^★★^	31.1 ± 2.9 ^★★^
NC	7	4.4 ± 0.5	4.7 ± 0.8	4.5 ± 0.6	4.8 ± 0.9	4.6 ± 0.9

The data are expressed as means ± SD. The differences between these groups and the DC group were statistically significant (denoted by “*” for *p* < 0.05, “**” for *p* < 0.01); the differences between the HD group and other groups were statistically significant (denoted by “^★^” for *p* < 0.05, “^★★^” for *p* < 0.01).

**Table 4:** Changes in random blood glucose level.

Groups	*n*	Random Blood Glucose (mmol/L)				
		Week 0	Week 1	Week 2	Week 3	Week 4
HD	7	31.2 ± 2.2	29.4 ± 2.3 *	25.3 ± 3.2 **	21.4 ± 3.6 **	18.2 ± 4.2 **
MD	7	31.1 ± 1.3	29.3 ± 2.5 *	26.4 ± 3.5 **	23.4 ± 2.1 **	21.3 ± 3.4 **^,^^★^
LD	7	31.4 ± 0.9	30.7 ± 2.3	28.5 ± 3.5 *^,^^★^	26.6 ± 3.2 **^,^^★^	24.9 ± 2.5 **^,^^★★^
BCA	7	31.6 ± 1.5	30.10 ± 1.2	28.2 ± 1.5 *^,^^★^	26.5 ± 2.5 **^,^^★^	24.2 ± 2.5 **^,^^★★^
CrT	7	31.1 ± 1.3	30.3 ± 1.5	27.4 ± 2.4 *	25.1 ± 1.7 **^,^^★^	22.2 ± 2.5 **^,^^★^
PC	7	31.2 ± 1.2	29.1 ± 1.5 *	25.2 ± 2.3 **	21.1 ± 1.8 **	17.1 ± 3.2 **
DC	7	31.4 ± 1.1	32.7 ± 1.6 ^★^	33.1 ± 0.8 ^★★^	32.8 ± 1.5 ^★★^	32.9 ± 1.1 ^★★^
NC	7	5.4 ± 0.4	5.8 ± 0.7	6.5 ± 1.2	6.2 ± 0.8	5.8 ± 0.6

The data are expressed as means ± SD. The differences between these groups and the DC group were statistically significant (denoted by “*” for *p* < 0.05, “**” for *p* < 0.01); the differences between the HD group and other groups were statistically significant (denoted by “^★^” for *p* < 0.05, “^★★^” for *p* < 0.01).

**Table 5:** Effect of [CrBCA_3_] on serum biochemical indices in mice.

	NC Group	DC Group	CrT Group	PC Group	HD Group	MD Group	LD Group	BCA Group
TC (mmol/L)	5.21 ± 0.24 **	8.35 ± 0.36 ^△△^	6.96 ±0.24^△^^,^*	5.92 ± 0.51 **	6.09 ± 0.28 **	7.15 ± 0.68 ^△^^,^*	7.96 ± 0.71 ^△△^	7.27 ± 0.59 ^△^^,^*
TG (mmol/L)	1.19 ± 0.18 **	2.83 ± 0.38 ^△△^	2.58 ± 0.45 ^△△^	2.21 ± 0.31 ^△△^^,^*	2.08 ± 0.46 ^△△^^,^*	2.56 ± 0.39 ^△△^	2.68 ± 0.42 ^△△^	2.55 ± 0.39 ^△△^
HDL-C (mmol/L)	1.62 ± 0.21 **	0.68 ± 0.12 ^△△^	0.96 ± 0.13 ^△△^^,^*	1.21 ± 0.16 ^△^^,^**	1.32 ± 0.21 ^△^^,^**	1.01 ± 0.22 ^△△^^,^*	0.85 ± 0.22 ^△△^^,^*	0.92 ± 0.14 ^△△^^,^*
LDL-C (mmol/L)	3.59 ± 0.23 **	5.16 ± 0.31 ^△△^	4.73 ± 0.42 ^△^	4.05 ± 0.26 ^△^^,^*	4.13 ± 0.32 ^△^^,^*	4.69 ± 0.29 ^△^	5.08 ± 0.41 ^△△^	4.61 ± 0.32 ^△^
GSP (mmol/L)	3.09 ± 0.23 *	3.92 ± 0.26 ^△^	3.46 ± 0.35 ^△^^,^*	3.25 ± 0.29 *	3.13 ± 0.36 *	3.45 ± 0.32 ^△^^,^*	3.71 ± 0.43 ^△^	3.62 ± 0.42 ^△^
NEFA (mmol/L)	2.15 ± 0.23 **	3.76 ± 0.31 ^△△^	3.14 ± 0.42 ^△△^^,^*	2.82 ± 0.26 ^△^^,^*	2.75 ± 0.21 ^△^^,^*	3.11 ± 0.28 ^△△^^,^*	3.66 ± 0.51 ^△△^	3.25 ± 0.36 ^△△^^,^*

The data are expressed as means ± SD, *n* = 7. The differences between these groups and the NC group were statistically significant (denoted by “^△^” or “^△△^” for *p* < 0.05 or *p* < 0.01, respectively); the differences between these groups and the DC group were statistically significant (denoted by “*” or “**” for *p* < 0.05 or *p* < 0.01, respectively).

**Table 6:** The effects on MDA levels and antioxidant enzyme activities in liver of db/db mice.

	NC Group	DC Group	CrT Group	PC Group	HD Group	MD Group	LD Group	BCA Group
MDA (nmol/mg)	3.25 ± 0.41 **	4.87 ± 0.62 ^△△^	4.19 ± 0.44 ^△^^,^*	3.59 ± 0.43 ^△^^,^**	3.66 ± 0.41 ^△^^,^**	3.98 ± 0.42 ^△^^,^*	4.52 ± 0.51 ^△△^	4.21 ± 0.53 ^△^^,^*
CAT (U/mg)	43.18 ± 3.28 **	21.42 ± 2.15 ^△△^	28.37 ± 2.56 ^△△^^,^*	37.34 ± 3.25 ^△^^,^**	35.43 ± 3.38 ^△^^,^**	30.23 ± 0.48 ^△△^^,^**	24.31 ± 0.44 ^△△^	28.43 ± 0.26 ^△△^^,^*
SOD (U/mg)	329.47 ± 9.23 **	232.48 ± 7.46 ^△△^	265.47 ± 7.39 ^△△^^,^*	301.42 ± 8.65 ^△^^,^**	297.58 ± 5.89 ^△^^,^**	271.44 ± 6.21 ^△△^^,^*	255.47 ± 6.82 ^△△^	260.58 ± 6.79 ^△△^^,^*
GSH-Px (U/mg)	621.45 ± 7.39 **	424.56 ± 7.56 ^△△^	512.51 ± 8.67 ^△△^^,^**	602.47 ± 6.25 **	593.39 ± 8.21 ^**^	524.72 ± 7.85 ^△△^^,^**	481.23 ± 8.83 ^△△^^,^*	506.28 ± 6.28 ^△△^^,^**

The data are expressed as means ± SD, *n* = 7. The differences between these groups and the NC group were statistically significant (denoted by “^△^” or “^△△^” for *p* < 0.05 or *p* < 0.01, respectively); the differences between these groups and the DC group were statistically significant (denoted by “*” or “**” for *p* < 0.05 or *p* < 0.01, respectively).

**Table 7:** Organ indices of mice in each group (means ± SD, *n* = 10).

	Liver	Kidney	Pancreas	Heart	Thymus	Spleen
TC group (mg/g)	61.47 ± 4.22	7.24 ± 0.81	5.38 ± 0.66	6.29 ± 0.59	2.24 ± 0.27	3.56 ± 0.36
NC group (mg/g)	61.21 ± 3.14	7.05 ± 0.69	5.02 ± 0.61	6.11 ± 0.68	2.12 ± 0.31	3.21 ± 0.41

**Table 8:** Effects of [CrBCA_3_] on lipid profiles, MDA levels and antioxidant enzyme activities of normal mice (means ± SD, *n* = 10).

Parameter	Normal Control Group (NC)	Toxicity Group (TC)
RBG (mmol/L)	6.14 ± 0.89	6.25 ± 0.96
INS (mIU/L)	11.12 ± 1.16	11.34 ± 1.38
TC (mmol/L)	5.23 ± 0.67	5.41 ± 0.58
TG (mmol/L)	1.21 ± 0.23	1.14 ± 0.21
LDL-C (mmol/L)	3.54 ± 0.21	3.65 ± 0.32
HDL-C (mmol/L)	1.71 ± 0.25	1.81 ± 0.23
GSP (μmol/L)	2.89 ± 0.25	2.95 ± 0.31
NEFA (μmol/g)	1.96 ± 0.14	2.14 ± 0.23
MDA (nmol/mg)	3.22 ± 0.35	3.14 ± 0.29
CAT (U/mg)	43.28 ± 5.11	44.25 ± 4.43
SOD (U/mg)	328.43 ± 13.47	326.45 ± 15.78
GSH-Px (U/mg)	625.37 ± 12.43	634.59 ± 12.47

**Table 9:** Effects of [CrBCA_3_] on blood routine and blood biochemical indices in sub-acute toxicity test (means ± SD, *n* = 10).

Parameter	Normal Control Group (NC)	Toxicity Group (TC)
WBC (10^9^/L)	9.71 ± 0.82	9.85 ± 0.96
RBC (10^12^/L)	7.53 ± 0.68	7.46 ± 0.84
HGB (g/L)	144.28 ± 9.21	149.88 ± 9.08
PLT (10^9^/L)	628.41 ± 51.26	633.48 ± 62.47
ALT (U/L)	39.59 ± 0.96	41.36 ± 0.92
AST (U/L)	142.58 ± 2.66	137.89 ± 2.98
ALP (U/L)	97.58 ± 1.15	95.23 ± 1.35
TP (g/L)	70.82 ± 0.96	72.55 ± 1.19
ALB (g/L)	31.46 ± 0.96	32.44 ± 1.01
BUN (mmol/L)	6.28 ± 0.29	6.13 ± 0.21
CR (μmol/L)	41.26 ± 1.35	42.58 ± 1.62

## Data Availability

All data generated or analyzed during this study are included in this published article.
